# Instrumental variable specifications and assumptions for longitudinal analysis of mental health cost offsets

**DOI:** 10.1007/s10742-012-0097-7

**Published:** 2012-09-25

**Authors:** A. James O’Malley

**Affiliations:** Department of Health Care Policy, Harvard Medical School, 180 Longwood Avenue, Boston, MA 02115-5899 USA

**Keywords:** Causal inference, Exclusion restriction, Fixed differences, Instrumental variable, Longitudinal, Mental health costs

## Abstract

Instrumental variables (IVs) enable causal estimates in observational studies to be obtained in the presence of unmeasured confounders. In practice, a diverse range of models and IV specifications can be brought to bear on a problem, particularly with longitudinal data where treatment effects can be estimated for various functions of current and past treatment. However, in practice the empirical consequences of different assumptions are seldom examined, despite the fact that IV analyses make strong assumptions that cannot be conclusively tested by the data. In this paper, we consider several longitudinal models and specifications of IVs. Methods are applied to data from a 7-year study of mental health costs of atypical and conventional antipsychotics whose purpose was to evaluate whether the newer and more expensive atypical antipsychotic medications lead to a reduction in overall mental health costs.

## Introduction

Estimation of causal effects in observational studies is an engrossing and controversial topic in statistics and the social sciences. Some investigators consider observational studies to lack internal validity as the absence of randomization exposes results to bias from unmeasured confounding variables. Yet observational studies are an important part of medical and health care research. They can be performed in situations where randomized trials are infeasible, they generate larger datasets, and they may involve more diverse study populations. Therefore, observational studies allow estimates of treatment effects for more nuanced subpopulations and are better equipped to account for treatment effect-heterogeneity than randomized trials.

Instrumental variables (IV) identify randomized experiments that are naturally occurring, enabling estimation of causal effects in observational studies. Loosely-speaking, an IV must predict treatment but not be directly related to the outcome or any unmeasured confounding variables (Imbens and Angrist 1994; Angrist et al. 1996). An IV extracts the variation in the supposed endogeneous predictor(s) that is orthogonal to any unmeasured confounding variables, yielding projected values from which the causal effect of the endogeneous predictor(s) on the outcome can be determined. Unlike regression and propensity score methods (Rosenbaum and Rubin 1983), IV methods accommodate unmeasured confounders. Although testing whether a variable predicts treatment is straight-forward, the requirement that the same variable does not directly affect the outcome (the *exclusion restriction*) is the bane of all IV analyses. First, even a small direct effect on the outcome violates the exclusion restriction. Second, it is not possible to test the exclusion restriction by simply including the IV as a predictor of the outcome as its own effect is then confounded with that of any unmeasured confounders (Morgan and Winship 2007, pp 196–197). Therefore, the choice of IVs must be undertaken with great care.

Longitudinal studies generalize cross-sectional designs by accommodating repeated observations over time on the same study unit (e.g., a patient). They allow dynamic treatment effects (e.g., the effect of a change in treatment) and modifying effects (e.g., the effect of current treatment changes with past treatment) to be estimated. In addition, individual dummy variables may be used to block the effects of time-invariant confounders. An important question is whether longitudinal data enhances examination of the IV assumptions.

In this paper we discuss the use of IVs in longitudinal analyses with particular focus on lagged predictors and outcomes. Treatment is represented using contemporaneous, lagged, and modifying variables. Because lagged treatment may be assumed to be endogeneous, exogeneous, an IV, or to have no effect whatsoever and lagged outcomes may be predictors, a multitude of longitudinal models are possible.

Various model specifications are compared by evaluating the effect of atypical versus conventional antipsychotic drugs on overall mental health costs defined as the cost of treatment and subsequent medical care in that year for medicaid recipients. The same data was analyzed previously by fitting a cross-sectional model using ordinary least squares (OLS) and various IV methods (O’Malley et al. 2011). However, in this cross-sectional setting the IV was borderline weak. Therefore, another key question is whether availability of longitudinal data allows the IV to be strengthened.

There are several important papers on IV methods for longitudinal (Hogan and Lancaster 2004; McClellan and Newhouse 1997) and other data types involving lagged variables, including spatially lagged data (Haining 1978; Kelejian and Robinson 1993). However, while several areas of statistical methodology consider the use of lagged variables as predictors (e.g., longitudinal analysis, time series analysis), their use as IVs has been studied less extensively. An exception is the work of several econometricians on methods for analyzing panel data (Arellano and Honore 2001, chapter 53; Hsiao 2003, chapter 4).

In Sect. 2 we review past work on mental health cost offsets and introduce the data and key variables motivating this work. The implication of differing assumptions about the causal relationships involving unmeasured confounding variables is illustrated using directed acyclic graphs (DAGs) in Sect. 3. In particular, we describe situations where lagged outcomes and treatments have different roles including when they should not be adjusted for, when they should be adjusted for, and when there is ambiguity. In Sect. 4 we introduce notation, models, estimands and IV assumptions for the mental health cost offsets analysis. Section 5 describes the IV requirements for each model and the method of estimation. In Sect. 6 we compare results across the models. The paper concludes with a discussion of the main findings in Sect. 7.

## Background

### Mental health cost offsets hypothesis

Atypical antipsychotics, including clozapine, olanzapine (zyprexa), quetiapine (sero-quel), and risperidone (risperidal), while considerably more expensive than the D2-antagonists, have been associated with a different (neurological versus physical) profile of side effects (O’Malley et al. 2011). It is thought that the greater tolerability of these new antipsychotics improves adherence to treatment regimens, thereby reducing relapses, resulting in declines in the use of hospital and emergency room services. This has led to the *offset hypothesis* that atypical antipsychotics, while more expensive ultimately *pay for themselves* through reductions in other types of health spending (Lichtenberg 2001). However, the hypothesis is disputed (Rosenheck et al. 2006) and testing it is complicated by the fact that patients who receive the newer atypical drugs likely differ from those getting the older drugs on a number of systematic factors, some unobserved.

### Study population and variables

The data motivating this research is from Florida’s Medicaid population over the period July 1994–June 2001. Study years are from July 1 of 1 year to June 30 of the next year. The analysis sample was restricted to patients continuously-enrolled for 6-months or more of a given study year (26,759 individuals).

Log-annual mental health spending is the dependent variable and plurality drug type (defined as a binary variable indicating whether atypical or conventional antipsychotic drugs comprised the majority of an individual’s Medicaid claims for the year) is the key predictor or “treatment.” The assumed exogeneous predictors are male, white, black, history of substance abuse, recipient of supplemental security income (SSI), study year and area of residence. Because Miami–Key West is the most populous area, indicator variables for the ten other areas are included as predictors. Unmeasured confounders could include health status of the patient (other physical and mental health comorbidities, severity of illness), patient preferences over treatment, access to skilled physicians, and physician prescribing habits. Many of these are time-varying and therefore cannot be blocked by patient dummies.

The approval status of the atypicals introduced during our study period—zyprexa, seroquel, geodon—and their interactions with area of residence were previously used as IVs. 1 Clearly, whether a drug has been approved impacts the likelihood an individual receives an atypical at a given time. Because areas have different geographic, cultural, social and economic factors and physicians in them may have varying attitudes, the uptake of atypicals is likely to vary between areas. Thus, the likelihood a patient is prescribed an atypical is expected to depend on where they live (O’Malley et al. 2011). In this paper the consequence of supplementing these IVs with additional variables only available with longitudinal data will be investigated.

In cross-sectional analyses emulating those conducted previously, OLS regression obtained an estimate of 1.022 (*P* < 0.0001), indicating that atypicals are much more expensive, while the two-stage least squares (2SLS) estimate was −0.028 (*P* = 0.866) (Table 1). However, the *F*-statistic of the Stock-Yogo (2002) test of a weak instrument was 9.69, just below the 10 % threshold (11.28) for rejecting the hypothesis that the IVs are weak.

**Table 1:** Basic identification improvements over cross-sectional analysis

Model	Estimate	*t*-stat	*P*-value	F_StageI_
Ordinary least squares				
Cross-sectional	1.022	76.4	0.000	
Fixed differences	0.613	44.1	0.000	
IV regression (two-stage least squares)				
Cross-sectional	−0.028	−0.17	0.866	9.69
Fixed differences	−0.590	−3.46	0.001	7.31
Add *a*_*i*(*t*-2)_ as IV	**0.133**	**1.28**	**0.199**	**15.5**

*F*_StageI_ denotes the Cragg-Donald *F*-statistic for the weak IV test for *a*_*it*_. The Stock-Yogo 10 and 20 % critical values with 35 exogeneous predictors (excluded instruments) are 11.26 and 6.03 respectively. *F*_StageI_ exceeds the 10 % threshold when *a*_*i*(*t*−2)_ is an IV and the first-difference method is used for estimation. Well-identified IV results appear in bold

## Causal assumptions

Conditioning on different subsets of the history of the outcomes or the treatments has been shown to have dramatic effects on the resulting inference (Pepe and Anderson 1994; Vansteelandt 2007). Therefore, it is important to consider the implications of including or excluding each candidate predictor in the model. DAGs are useful for depicting the data generating mechanism and the causal assumptions made by various models. Let *Y*, *A*, *X*, *U* and *Z* be random variables denoting the outcome, treatment, exogeneous covariates, unmeasured covariates, and IVs for an individual. We use the subscript *t* for time and for illustration consider the case $t \in \{0,1\}$.

### Conditioning on lagged treatments and outcomes

**Fig. 1:**
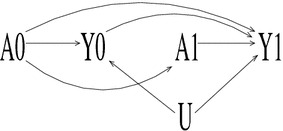
Directed acyclic graph (DAG) of a scenario where lagged treatment *A*_0_ must be conditioned on to identify the effect of *A*_1_ on the outcome *Y*_1_. However, lagged outcome *Y*_0_ is a common effect (or collider) for *A*_0_ and *U* and so conditioning on *Y*_0_ confounds the direct effect of *A*_0_ on *Y*_1_. Effect directionality is depicted by an *arrow*; absence of an *arrow* implies no effect

Figure 1 depicts a scenario where an unmeasured variable *U* affects *Y*_1_ and *Y*_0_ (i.e., the effect of *U* endures over time) but does not influence treatment selection at any point (*A*_1_ or *A*_0_). Furthermore, the outcome from one year does not influence treatment in the next. The DAG in Fig. 1 might arise when treatment is determined purely on the basis of a patient’s medical condition, implying previous years cost of treatment would not be expected to have any impact on subsequent treatment.

In order to obtain a consistent estimate of the effect of *A*_1_ on *Y*_1_, it is necessary to condition on *A*_0_ as it would otherwise be an unmeasured confounder. However, while conditioning on *Y*_0_ does not affect the identifiability of the effect of *A*_1_ on *Y*_1_, it has implications for the effect of *A*_0_ on *Y*_1_. If *Y*_0_ is not conditioned on then the direct effect of *A*_0_ on *Y*_1_ is confounded with the effect acting through *Y*_0_. If *Y*_0_ is conditioned on then the unblocked path from *A*_0_ to *Y*_1_ through *U* that arises as *Y*_0_ is caused by both *A*_0_ and *U* leads to lack of identifiability (Sharkey and Elwert 2010).^2^ Specifically, one cannot distinguish the effect of *A*_0_ on *Y*_1_ from that induced through *U*. Therefore, whether or not *Y*_0_ is conditioned on, the direct effect of *A*_0_ on *Y*_1_ is not-identified.

**Fig. 2:**
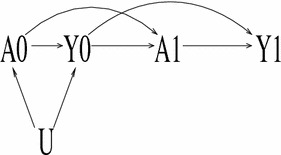
DAG of a scenario where it is necessary to condition on lagged outcome *Y*_0_ but not necessary to condition on lagged treatment *A*_0_. If *Y*_0_ is conditioned on, *A*_0_ becomes an IV for *A*_1_

Figure 2 depicts a different situation. The unmeasured variable *U* acts entirely in the past (e.g., a short-term external shock that affected preferences for and cost of atypicals at *t* = 0 only), *Y*_0_ affects *A*_1_ (e.g., patients switch to conventionals because they could not sustain the high copayments), and *A*_0_ does not directly affect *Y*_1_. Then *U* is a confounder of the effect of *A*_0_ on *Y*_0_ but does not directly cause *A*_1_ or *Y*_1_.^3^ Because *Y*_0_ is a cause of *Y*_1_ and *A*_1_, failing to adjust for *Y*_0_ results in the unmeasured confounding at *t* = 0 transferring to *t* = 1. Therefore, adjusting for *Y*_0_ is necessary in order to block *U*. However, because *Y*_0_ blocks all backdoor pathways from *A*_1_ to *Y*_1_, it is not necessary to also condition on *A*_0_, which could function as an IV. If the arrow from *Y*_0_ to *A*_1_ didn’t exist, *U* can be blocked by conditioning on *A*_0_ or *Y*_0_.

### Need for IVs

**Fig. 3:**
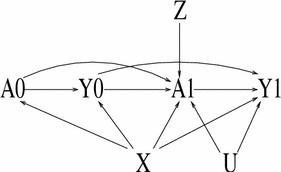
DAG of a scenario where an instrumental variable *Z* identifies the effect of *A*_1_ on *Y*_1_ without needing to conditioning on any other variables. However, if *Z* was also a cause of *A*_0_ (an *arrow* from *Z* to *A*_0_ is added to the DAG), the only way that *Z* remains a valid IV for *A*_1_ is by conditioning on the observed covariates *X* and either *Y*_0_ or *A*_0_. If *X* and *Y*_0_ are conditioned on then *A*_0_ is an additional IV for the effect of *A*_1_ on *Y*_1_

Figure 3 depicts a situation like Fig. 2 except that *U* confounds the effect of *A*_1_ on *Y*_1_ as opposed to that of *A*_0_ on *Y*_0_. Because there is no way to block the path from *A*_1_ to *Y*_1_ through *U*, the only way that a causal estimate can be recovered is to use the IV *Z* to isolate the variation in *A*_1_ that is independent of *U*. A simple check of the validity of *Z* as an IV is that it be on a path into *A*_1_ but not be on any path into *Y*_1_ that does not pass through *A*_1_.

Under Figure 3 the IV analysis does not need to involve *Y*_0_, *A*_0_ or *X*. However, if *Z* also caused *A*_0_ it would then be necessary to condition on *X* and either *Y*_0_ or *A*_0_. Because conditioning on *Y*_0_ and *X* blocks all paths from *A*_0_ to *Y*_1_, a test of the validity of the model assumptions is to include *A*_0_ in the model; a statistically significant coefficient of the effect of *A*_0_ on *Y*_1_ would raise concerns about the validity of the model.^4^

## Notation and models for offsets analysis

Let *y*_*it*_, *a*_*it*_, ***x***_*it*_, *u*_*it*_ and *z*_*it*_ denote *Y*, *A*, *X*, *U*, and *Z* respectively for individual *i* in study-year *t*. Treatment is coded *a*_*it*_ = 1 for atypicals and *a*_*it*_ = 0 for conventionals. The cross-sectional model assumed in O’Malley et al. (2011) is given by

1 $$ y_{i*} = \beta_{1}a_{i*} + {\varvec{\beta}}_{2}^{T}{\user2{x}}_{i*} + \epsilon_{i*}, $$

where the index i* is used to emphasize that in this model we ignore the fact that some subjects contribute observations to multiple years. Unlike regular regression models, the model in (1) allows corr $(a_{i*},\epsilon_{i*}) \neq 0$. It provides a baseline for demonstrating how longitudinal data may enrich IV analyses (see Sect. 6.1).

### Longitudinal models

**Fig. 4:**
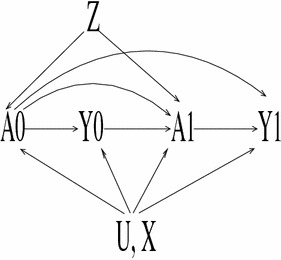
DAG for instrumental variables analysis when the IVs *Z*, the observed covariates *X* (including the dummy variables for each region), and the unmeasured covariates *U* have lagged and contemporaneous effects. Because it is necessary to instrument for both *A*_0_ and *A*_1_, *Z* must have dimension ≥ 2. If the outcome *Y* was serially dependent (an *arrow* from *Y*_0_ to *Y*_1_ is added to the DAG) then *Z* would not be a valid IV for (*A*_0_,*A*_1_); conditioning on *Y*_0_ blocks *A*_0_ at the expense of opening the backdoor path through *U*

With individuals in the offsets data observed for up to seven years, a plethora of lagged variables may be predictors. We focus on only models with single-lagged variables as predictors. Although we perform some analyses excluding *y*_*i*(*t*−1)_ and *a*_*i*(*t*−1)_, for brevity we only present mathematical model specifications with them included. To emphasize that different models are identifiable under different assumptions about *u*_*it*_, Figure 4 presents a scenario under which *y*_*i*(*t*−1)_ (depicted by *Y*_0_) must not be conditioned on in order for the IV to identify the effects of (*a*_*i*(*t*−1)_,*a*_*it*_) (depicted by (*A*_0_,*A*_1_) on *y*_*it*_ (depicted by *Y*_1_). The key identifiability condition under this DAG is that *y*_*i*(*t*−1)_ not affect *y*_*it*_. If *y*_*i*(*t*−1)_ affects *y*_*it*_ then an alternative exclusion restriction is needed; for example, the condition that the unmeasured variable *u*_*it*_ (depicted by *U*) has no effect on *y*_*i*(*t*−1)_ would suffice.

The treatment variables may also include *a*_*it*_ *a*_*i*(*t*-1)_ and interactions with the elements of ***x***_*it*_, although we do not consider the latter here. The terms “dynamic-treatment model” and “modified-treatment model” refer to the models given by

2 $$ y_{it} = \beta_{0i} + \beta_{1} y_{i(t-1)} + \beta_{2}a_{it} + \beta_{3}a_{i(t-1)} + {\varvec{\beta}}_{5}^{T}{\user2{x}}_{it} + \beta_{5}u_{it} + \epsilon_{it} $$

and

3 $$ y_{it} = \beta_{0i} + \beta_{1} y_{i(t-1)} + \beta_{2}a_{it} + \beta_{3}a_{i(t-1)} + \beta_{4}a_{i(t-1)}a_{it} + {\varvec{\beta}}_{5}^{T}{\user2{x}}_{it} + \beta_{6}u_{it} + \epsilon_{it} $$

respectively, where * β*_0*i*_ is an individual-specific effect that accounts for all time invariant effects. The lagged outcome *y*_*i*(*t*−1)_ and lagged treatment *a*_*i*(*t*−1)_ absorb time-varying effects that acted prior to time period *t*.

Inclusion of *a*_*it*_ *a*_*i*(*t*−1)_ as an additional predictor in (3) allows for the effect of continued treatment on an atypical to differ from the sum of its contemporaneous and lagged effects. Equating coefficients with those in the following alternative specification of (3),

$$ y_{it} = \beta_{0i} + \beta_{1} y_{i(t-1)} + \tilde{\beta}_{2}(1-a_{i(t-1)})a_{it} + \tilde{\beta}_{3}a_{i(t-1)}(1-a_{it}) + \tilde{\beta}_{4}a_{i(t-1)}a_{it} + {\varvec{\beta}}_{5}^{T}{\user2{x}}_{it} + \beta_{6}u_{it} + \epsilon_{it}, $$

it follows that $\tilde{\beta}_{2}=\beta_{2}, \; \tilde{\beta}_{3}=\beta_{3}$, and $\tilde{\beta}_{4}=\beta_{2}+\beta_{3}+\beta_{4}$. Therefore, relative to continued use of a conventional, *β*_2_ is the effect of switching from a conventional to an atypical, *β*_3_ is the effect of switching from an atypical to a conventional, and *β*_2_ + *β*_3_ + *β*_4_ is the effect of staying on an atypical throughout. If *β*_3_ + *β*_4_ > 0 then the expected total cost of mental health care for the year is greater if an individual took an atypical in the prior year than if they are a new atypical prescriber. If atypicals have higher upfront costs and lower costs thereafter, one would instead expect *β*_3_ + *β*_4_ < 0.

If *a*_*it*_ is endogeneous then any variable that interacts with *a*_*it*_ is also endogeneous. However, while *a*_*it*_ *a*_*i*(*t*−1)_ inherits endogeneity from *a*_*it*_, *a*_*i*(*t*−1)_ need not be endogeneous. For both (2) and (3) we evaluate the consequence of *a*_*i*(*t*−1)_ endogeneous (as in Fig. 4), exogeneous (as in Fig. 1), and usable as an IV (as in Fig. 2 or Fig. 3). Because adjusting for *y*_*i*(*t*−1)_ can be problematic (Figs. 1, 4), the estimates obtained under this model are compared to those for models that exclude *y*_*i*(*t*−1)_.

Although random effect models are common in longitudinal analyses they are problematic when *y*_*i*(*t*−1)_ (or other lagged outcome) is a predictor as the assumption that random *β*_0*i*_ is uncorrelated with the predictors is violated (Wooldridge 2002, p. 256). This is seen from the fact that *β*_0*i*_ affects the expected value of all observations on an individual, including *y*_*i*(*t*−1)_. Therefore, under a random effects specification, *β*_0*i*_ would be correlated with *y*_*i*(*t*−1)_, which is a predictor of *y*_*it*_. Thus, we avoid random effect specifications for *β*_0*i*_. Because we don’t model the correlation structure we use robust standard errors to account for dependence within individuals (Huber 1967; White 1982).

## IV requirements

The general requirements for ***z***_*it*_ to be an IV for the effect of *a*_*it*_ on *y*_*it*_ are: (1) it is associated with *a*_*it*_ conditional on ***x***_*it*_, *u*_*it*_; (2) it is not associated with *u*_*it*_ conditional on ***x***_*it*_; (3) it is not associated with *y*_*it*_ conditional on *a*_*it*_, ***x***_*it*_, *u*_*it*_. The more precisely ***z***_*it*_ predicts *a*_*it*_ the greater the statistical power of the analysis; perfect predictions typically occur only in randomized studies with 100 % compliance with treatment assignment. Condition (2) guards against any backdoor pathways from ***z***_*it*_ through *u*_*it*_ to *y*_*it*_—sometimes referred to as the “random” requirement. Condition (3) excludes ***z***_*it*_ from having a direct effect on *y*_*it*_ other than through *a*_*it*_—the “exclusion restriction.”

A DAG-based test of ***z***_*it*_ as an IV in Fig. 4 is: after removing all arcs out of *a*_*it*_ no path leads from ***z***_*it*_ to *y*_*it*_ conditional on ***x***_*it*_ (Brito and Pearl 2002; Joffe et al. 2008). Any unmeasured area level variables are absorbed in *u*_*it*_. However, because such variables are time-invariant the inclusion of the area dummies in ***x***_*it*_ blocks their effects.

### Using longitudinal data to enhance IVs

In the cross-sectional analysis of the offsets data, the IVs were contemporaneous indicators of the approval status of zyprexa, seroquel and geodon and their interactions with area of residence. However, the model for the outcome is suggestive of additional IVs; {*a*_*i*(*t*−*k*)_}_*k*>1_ do not appear in either (2) or (3), which is consistent with them not having a direct effect on *y*_*it*_. Because *a*_*i*(*t*−2)_ is evaluated at least a year earlier than *y*_*it*_, it is plausible that it is uncorrelated with *y*_*it*_ conditional on (*y*_*i*(*t*−1)_, *a*_*it*_, *a*_*i*(*t*−1)_, *x*_*it*_). If *a*_*i*(*t*−2)_ is correlated with *a*_*it*_ conditional on (*a*_*i*(*t*−1)_, *x*_*it*_, *u*_*it*_) then *a*_*i*(*t*−2)_ is a valid IV. In general, if treatment influences subsequent treatment for a longer period than it influences outcomes, then the lagged treatment variables from the differential period are candidate IVs.^5^

When β_3_ = 0 in (2), *a*_*i*(*t*−1)_ is a candidate IV for *a*_*it*_. However, if *a*_*i*(*t*−1)_ is associated with an unmeasured confounder (e.g., as in Fig. 1 when *Y*_0_ is conditioned on), it violates the IV assumptions. If *a*_*i*(*t*−2)_ or any other variable is known to be a valid IV, the Sargan over-identifying restrictions test (ORT) may be used to evaluate whether *a*_*i*(*t*−1)_ is a valid IV (Sargan 1958).

### Estimation: two-stage least squares (2SLS)

To avoid estimating the fixed effects {*β*_0*i*_}_1:*N*_, estimation of the longitudinal models is accomplished by regressing the individually-first differenced outcomes on the individually-first-differenced predictors (Wooldridge 2002, pp. 279–281). Because differencing accounts for all time-invariant variation, the strength of the IV is governed by the extent to which intra-individual variation in ***z***_*it*_ predicts intra-individual variation in *a*_*it*_. Conversely, the exclusion restriction is only violated by intra-individual variation directly related to *y*_*it*_.

A virtue of first differencing over mean-centering (subtraction of the individual sample mean $\bar{v}_{i}$ from *v*_*it*_, $t=1,\ldots,T$) is that it makes *a*_*i*(*t*−2)_ more defensible as an IV. This is seen from that fact that under (2) and (3) the first-differenced error, $\epsilon_{it}-\epsilon_{i(t-1)}$, is independent of *a*_*i*(*t*−2)_ − *a*_*i*(*t*−3)_. However, if *a*_*it*_ depends on $\epsilon_{it}$ for *t* = 1,…,*T* then $a_{i(t-2)}-\bar{a}_{i}$ and the mean-centered error $\epsilon_{i(t-2)}-\bar{\epsilon}_{i}$ appear likely to be correlated.

By using *a*_*i*(*t*-2)_ as an IV and basing estimates on first-differences, only observations with non-missing (*a*_*it*_, *a*_*i*(*t*−1)_, *a*_*i*(*t*-2)_, *a*_*i*(*t*−3)_) are used in the analysis leading to a substantial loss of information. Rather than require that all IVs be available for all observations, we do not use *a*_*i*(*t*−2)_ as an IV for observations in which it is missing [an approach proposed in Arellano and Bond (1991)]. Let *r*_*it*_ = 1 if *a*_*i*(*t*−2)_ is missing and *r*_*it*_ = 0 otherwise. Then set the component of *z*_*it*_ corresponding to *a*_*i*(*t*−2)_ equal to 0 if *r*_*it*_ = 0. Because *r*_*it*_ is not expected to contain any information about *y*_*it*_ we use it as an additional IV. If all of the IVs are valid then the treatment effect is not affected by the removal or addition of any particular IV from the analysis (Small 2007). Therefore, using *r*_*it*_ as an additonal IV is only expected to affect the precision of the estimated treatment effects.

We illustrate the 2SLS estimation procedure for (2) in the case when *y*_*i*(*t*−1)_ and *a*_*i*(*t*−1)_ are conditioned on (an action consistent with the DAG in Fig. 3). Let $\tilde{v}_{it}=v_{it}-v_{i(t-1)}$. The 2SLS procedure is then:

1. Use OLS to fit the “stage I” regression equation
  $$ \tilde{a}_{it} = \theta_{1}\tilde{y}_{i(t-1)} + \theta_{2}\tilde{a}_{i(t-1)} + {\varvec{\theta}}_{3}^{T}{\tilde{\user2{x}}}_{it} + {\varvec{\theta}}_{4}^{T}{\tilde{\user2{z}}}_{it} + \tilde{\delta}_{it} $$
  to obtain fitted values $\hat{{a}}_{it}.$
2. Use OLS to fit the outcome or “stage II” regression equation
  $$ \tilde{y}_{it} = \beta_{1} \tilde{y}_{i(t-1)} + \beta_{2}\hat{{a}}_{it} + \beta_{3}\tilde{a}_{i(t-1)} + {\varvec{\beta}}_{5}^{T}{\tilde{\user2{x}}}_{it} + \tilde{\epsilon}_{it}, $$
  yielding estimates of β_2_ and the other model parameters.

As depicted above, all exogeneous predictors in the outcome (stage II) equation are included in the stage I equation (Angrist and Pischke 2009, p. 189).

When *a*_*i*(*t*−1)_ is endogeneous (e.g., if a time-varying unmeasured confounder exists), there are two endogeneous variables and thus two stage I equations. Because the stage I equations must include all the predictors in the outcome equation other than the endogeneous variables, $\hat{{a}}_{it}$ and $\hat{{a}}_{i(t-1)}$ are the fitted values of $\tilde{a}_{it}$ and $\tilde{a}_{i(t-1)}$ obtained from

4 $$ \begin{array}{cc} \tilde{a}_{it} &= \theta_{1,1}\tilde{y}_{i(t-1)} + {\varvec{\theta}}_{2,1}^{T}{\tilde{\user2{x}}}_{it} + {\varvec{\theta}}_{3,1}^{T}{\tilde{\user2{z}}}_{it} + \tilde{\delta}_{it,1} \\ \tilde{a}_{i(t-1)} &= \theta_{1,2}\tilde{y}_{i(t-1)} + {\varvec{\theta}}_{2,2}^{T}{\tilde{\user2{x}}}_{it} + {\varvec{\theta}}_{3,2}^{T}{\tilde{\user2{z}}}_{it} + \tilde{\delta}_{it,2}. \end{array} $$

If ***z***_*it*_ is a candidate IV for *a*_*it*_, ***z***_*i*(*t*−1)_ is a candidate IV for *a*_*i*(*t*−1)_. However, use of ***z***_*i*(*t*−1)_ as an IV in the offsets analysis had little impact on the results and, if anything, reduced the efficacy of the IV in the sense that the amount of variation explained per parameter estimated in the stage-I equation was substantially lower.

A curious feature of (4) is that $\tilde{y}_{i(t-1)}$, ${\tilde{\user2{x}}}_{it}$, and ${\tilde{\user2{y}}}_{it}$ are predictors of $\tilde{a}_{i(t-1)}$ (second equation). The anomaly that $\tilde{y}_{i(t-1)}$ is a predictor of $\tilde{a}_{i(t-1)}$ in (4) emphasizes that the stage I equations do not depict models that we believe in but are artifacts of the estimation procedure. The stage I equations are determined solely by the outcome equation and the designated instruments. In contrast, under a parametric structural equation model such as the “Heckit model” (Arendt and Holm 2008), a bivariate model is assumed in which the predictors in the treatment selection equations (for *a*_*it*_, *a*_*i*(*t*−1)_) need not include the same exogeneous predictors as the outcome equation for *y*_*it*_.

When *a*_*i*(*t*−1)_ is exogeneous, (3) utilizes two endogeneous predictors implying that the 2SLS procedure involves two stage I equations. If ***z***_*it*_ is an IV for *a*_*it*_, ***z***_*it*_ *a*_*i*(*t*−1)_ is a candidate IV for *a*_*it*_ *a*_*i*(*t*−1)_.^6^ We tested whether ***z***_*it*_ *a*_*i*(*t*−1)_ was a suitable IV but found it had minimal impact on the results. Therefore, the stage I equations for 2SLS are

5 $$ \begin{array}{cc} \tilde{a}_{it} &= \theta_{1,1}\tilde{y}_{i(t-1)} + \theta_{2,1} \tilde{a}_{i(t-1)} + {\varvec{\theta}}_{3,1}^{T}{\tilde{\user2{x}}}_{it} + {\varvec{\theta}}_{4,1}^{T}{\tilde{\user2{z}}}_{it} + \tilde{\delta}_{it,1} \\ {a_{it}\tilde{a}_{i(t-1)}} &= \theta_{1,2}\tilde{y}_{i(t-1)} + \theta_{2,2} \tilde{a}_{i(t-1)} + {\varvec{\theta}}_{3,2}^{T}{\tilde{\user2{x}}}_{it} + {\varvec{\theta}}_{4,2}^{T}{\tilde{\user2{z}}}_{it} + \tilde{\delta}_{it,2} \end{array} $$

In (3), if *a*_*i*(*t*−1)_ is endogeneous then three stage I equations are required and ***z***_*it*_ *a*_*i*(*t*−1)_ or any other interactions involving *a*_*i*(*t*−1)_ cannot be IVs.

The Stata procedure xtivreg2 with estimation option “fd” (for first differences) may be used to fit the longitudinal models described above. Example code is provided in the Appendix.

## Results

**Fig. 5:**
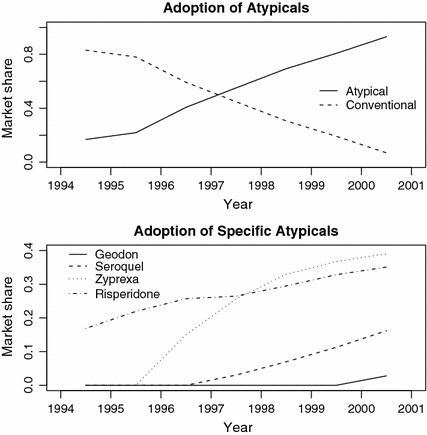
Share of antipsychotic market held by atypical and conventional drugs (*upper plot*) and specific atypical drugs (*lower plot*), 1994–2001

We examine the strength of the IVs by plotting adoption rates over time. The market share of atypicals increased dramatically over 1994–2001 (Fig. 5). Following the approval of zyprexa, the market share of atypicals increased more rapidly while the subsequent approval of seroquel and geodon maintained rather than accelerated the rate of increase. Nonetheless, the approval status of the atypicals is clearly associated with the likelihood a patient takes an atypical.

**Fig. 6:**
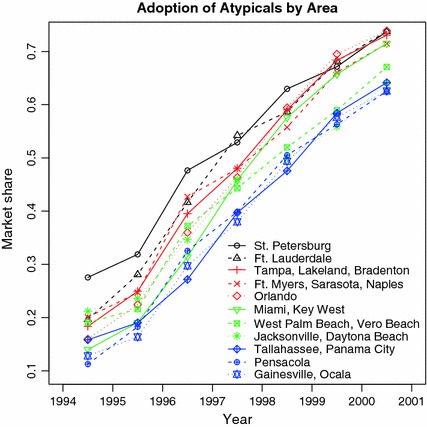
Share of antipsychotic market held by atypical and conventional drugs in 11 areas in Florida, 1994–2001. In the legend, the top-to-bottom ordering of the areas is by average decrease in market share over 1994–2001. Thus, St. Petersburg had the greatest spending on average and Gainesville–Ocala the least

Figure 6 reveals substantial and largely consistent differences in the rate of adoption or utilization of atypicals between areas (Fig. 6). For example, St Petersburg consistently had one of the highest market shares while Gainesville–Ocala consistently had one of the lowest. Because differences in adoption rates across areas are believed to not directly affect mental health care costs, the differential variation between areas can be used to help identify the effect of atypical use on mental health costs. Thus, dummy variables for approval status of zyprexa, seroquel and geodon and their interactions with area of residence are plausible IVs.

**Fig. 7:**
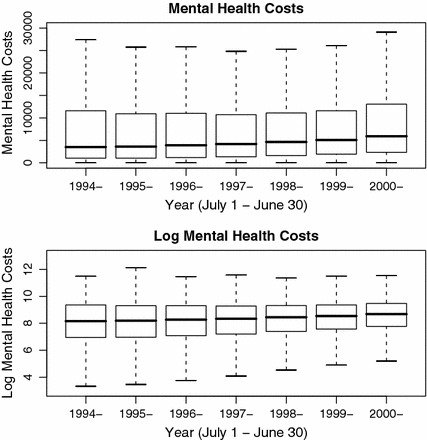
*Box* and *whisker* plots of the distribution of mental health costs, 1994–2001. The original and log-transformed costs appear in the upper and lower segments respectively. The five-number summaries are indicated by the horizontal lines and correspond to the (2.5,25,50,75,97.5)’th percentiles of the distribution of total mental health costs

**Fig. 8:**
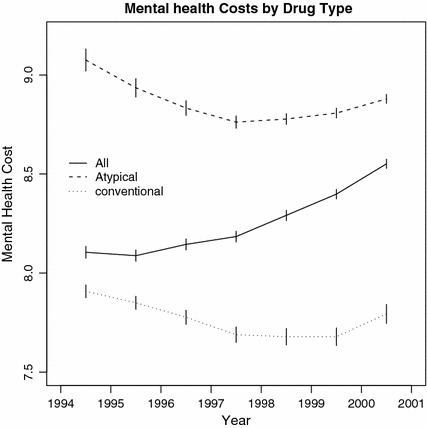
Unadjusted average annual mental health costs for atypicals and conventionals, 1994–2001. The increase in the average total annual mental health costs reflects the increased adoption and utilization of atypicals over 1994–2001

Average annual mental health costs increased over 1994–2001 (Fig. 7). The distribution of cost is skewed to the right whereas the distribution of log-cost is nearly symmetric, indicating the appropriateness of log-transformation. Figure 8 recapitulates that patient-year mental health costs have increased and also reveals that this is due to increasing market share of the more costly atypicals. Indeed, the trajectories of log-mental-health costs for atypical and conventional users are parallel and for the most part decreasing. Thus, it is an artifact of Simpson’s paradox that, due to the changing share of atypicals, overall mental health costs increased.

### Strengthening IV in cross-sectional model

The potential for longitudinal data to enhance IV estimation is first demonstrated by fitting the cross-sectional model in (1), then first-differencing to account for time-invariant confounders, and finally augmenting the IVs with *a*_*i*(*t*−2)_. The substantial difference between the OLS and 2SLS estimates of *β*_1_ under (1) can be attributed to extensive unmeasured confounding (Table 1). Although the effect of *a*_*i*(*t*−2)_ is reduced by first-differencing, the IV assumptions are more believable as time-invariant unmeasured variables are blocked. Despite only being identified off intra-individual variation, the doubling of the F_StageI_ statistic reveals that use of *a*_*i*(*t*−2)_ as an IV substantially improves identification of the effect of *a*_*it*_ on *y*_*it*_.

### Dynamic model

We consider the four models given by *y*_*i*(*t*−1)_ (included, excluded) and *a*_*i*(*t*−1)_ (included, excluded). In 2SLS analyses, two scenarios are considered when *a*_*i*(*t*−1)_ is included (endogeneous, exogeneous) and excluded (IV, not an IV) from the model. Throughout the longitudinal analyses *a*_*i*(*t*−2)_ is embedded in ***z***_*it*_. Unless otherwise stated, results pertain to the case when *y*_*i*(*t*−1)_ is excluded from the analysis.

The OLS results for the dynamic model reveal that atypicals are more costly (estimate 0.625, *P* < 0.001); and that there is a small carry-over effect of previous years atypical use (estimate 0.107, *P* < 0.001) (Table 2). Therefore, *a*_*it*_ is a more influential determinant of *y*_*it*_ than *a*_*i*(*t*−1)_. Inclusion of *y*_*i*(*t*−1)_ in the model has little impact on estimates under OLS.

**Table 2:** Longitudinal models with different roles of *a*_*i*(*t*−1)_: no treatment modification

Status	Term	*y*_*i*(*t*−1)_ Excluded				*y*_*i*(*t*−1)_ Included			
of *a*_*i*(*t*-1)_		Estimate	*t*-stat	*P*-value	*F*_StageI_	Estimate	*t*-stat	*P*-value	*F*_StageI_
Ordinary least squares									
Exogeneous	*a*_*it*_	0.625	37.7	0.000		0.622	40.2	0.000	
	*a*_*i*(*t*-1)_	0.107	7.57	0.000		0.288	20.7	0.000	
Exclude	*a*_*it*_	0.613	44.1	0.000		0.603	44.0	0.000	
IV regression (two-stage least squares)									
Endogeneous	*a*_*it*_	−0.686	−3.42	0.001	6.04	−0.997	−4.49	0.000	3.91
	*a*_*i*(*t*-1)_	0.374	5.53	0.000		0.601	7.58	0.000	
Exogeneous	*a*_*it*_	**0.355**	**6.51**	**0.000**	**54.5**	**0.218**	**4.20**	**0.000**	**54.5**
	*a*_*i*(*t*-1)_	**0.027**	**1.34**	**0.179**		**0.169**	**8.67**	**0.000**	
Instrument	*a*_*it*_	**0.297**	**8.91**	**0.000**	**142.7**	**−0.134**	**−3.98**	**0.000**	**136.6**
Exclude	*a*_*it*_	**0.133**	**1.28**	**0.199**	**15.5**	**0.403**	**4.21**	**0.000**	**14.0**

The Stock-Yoga *F*-test 5 and 10 % critical values for a single endogeneous predictor are approximately 21.4 and 11.26 respectively. Well-identified IV results appear in bold

Results under IV estimation are well identified when *a*_*i*(*t*−1)_ is used in some form to predict *a*_*it*_ in the stage I equation (F_StageI_ in excess of 50 as an exogeneous predictor and in excess of 100 as an IV), moderately well-identified if *a*_*i*(*t*−1)_ is excluded altogether (F_StageI_ around 15), and poorly-identified if *a*_*i*(*t*−1)_ is endogeneous. The level of identification is minimally affected by conditioning on *y*_*i*(*t*−1)_. The lack of identifiability in the endogeneous case is compounded by high colinearity between *a*_*it*_ and *a*_*i*(*t*−1)_, which even in the absence of unmeasured confounders makes it difficult to extract the independent effect of each and often increases the magnitude and alternates the signs of the predictors (as for the offsets analysis).

Because the inclusion of *y*_*i*(*t*−1)_ as a predictor impacts the results in different ways, the three “identified” cases are discussed each in turn. When *a*_*i*(*t*−1)_ is an exogeneous covariate the coefficient of *a*_*it*_ is significant and positive (estimate 0.0355, *P* < 0.001) while the coefficient of *a*_*i*(*t*−1)_ is not significantly different from 0. The inclusion of *y*_*i*(*t*−1)_ led to an increase in the effect of *a*_*i*(*t*−1)_ at the expense of the effect of *a*_*it*_. Although the estimate of *β*_2_ (the effect of *a*_*it*_) is bigger than *β*_3_ (the effect of *a*_*i*(*t*−1)_), the latter has a higher t-statistic due to the fact that it is not instrumented.

When *a*_*i*(*t*−1)_ is an IV there is only a minor change to the exogeneous case—a consequence of the estimated β_3_ being close to 0 when *a*_*i*(*t*−1)_ is a predictor. However, when *y*_*i*(*t*−1)_ is included, the estimate of β_2_ is negative and significant (estimate −0.134, *P* < 0.001). This is the only well-identified longitudinal specification under which atypicals appear to lower the cost of mental health care. However, one reason to doubt analyses with *a*_*i*(*t*−1)_ as an IV is that $\tilde{a}_{i(t-1)}=a_{i(t-1)}-a_{i(t-2)}$ and $\tilde{\epsilon}_{it}=\epsilon_{it}-\epsilon_{i(t-1)}$ seem likely to be correlated as endogeneous treatment assignment implies *a*_*i*(*t*−1)_ and $\epsilon_{i(t-1)}$ are correlated.

If *a*_*i*(*t*−1)_ is excluded altogether then β_2_ is estimated to be 0.133 (not significant) when *y*_*i*(*t*−1)_ is excluded and 0.403 (*P* < 0.001) when *y*_*i*(*t*−1)_ is included. Thus, the impact of *y*_*i*(*t*−1)_ is opposite that when *a*_*i*(*t*−1)_ is used as an IV. Unfortunately, it is not possible to test empirically whether conditioning on *y*_*i*(*t*−1)_ is more problematic than not conditioning on *y*_*i*(*t*−1)_. However, conditioning generally introduces less bias than not conditioning (Greenland 2003), suggesting that the results under the exogeneous specification might be the more trustworthy. Because the estimates of both β_2_ and β_3_ are positive and significant under the exogeneous specification, the offsets hypothesis appears to not hold.

### Modified-treatment model

The OLS results for the modified-treatment model (Table 3) suggest that *a*_*it*_ *a*_*i*(*t*−1)_ has a statistically significant positive effect (β_4_ > 0), implying that mental health costs of atypicals are greater when atypical use is continued from the year prior than when newly adopted. However, the effect of atypical use in the current year is larger than the modification for prior use. Because the main effect of *a*_*i*(*t*−1)_ is close to 0, the effect of atypical use appears to dissipate immediately upon stopping.

**Table 3:** Longitudinal models with different roles of *a*_*i*(*t*−1)_: treatment modification

Status	Term	*y*_*i*(*t*−1)_ Excluded				*y*_*i*(*t*−1)_ Included			
of *a*_*i*(*t*−1)_		Estimate	*t*-stat	*P*-value	*F*_StageI_	Estimate	*t*-stat	*P*-value	*F*_StageI_
Ordinary least squares									
Exogeneous	*a*_*it*_	0.635	34.0	0.000		0.624	36.7	0.000	
	*a*_*i*(*t*−1)_	−0.030	−1.04	0.299		−0.007	−0.25	0.800	
	*a*_*it*_ *a*_*i*(*t*−1)_	0.126	5.09	0.000		0.292	12.9	0.000	
Exclude	*a*_*it*_	0.608	43.7	0.000		0.593	43.2	0.000	
	*a*_*it*_ *a*_*i*(*t*−1)_	0.100	8.75	0.000		0.181	15.0	0.000	
Two-stage least squares									
Endogeneous	*a*_*it*_	−0.472	−2.74	0.006	2.22	−0.675	−3.76	0.000	2.21
	*a*_*i*(*t*−1)_	−0.398	−1.82	0.069		−0.499	−2.29	0.022	
	*a*_*it*_ *a*_*i*(*t*−1)_	1.106	3.16	0.002		1.55	4.41	0.000	
Exogeneous	*a*_*it*_	0.273	4.09	0.000	7.17	0.133	2.07	0.038	7.16
	*a*_*i*(*t*−1)_	0.863	3.64	0.000		0.930	4.10	0.000	
	*a*_*it*_ *a*_*i*(*t*−1)_	−0.476	−3.26	0.001		−0.370	−2.66	0.008	
Instrument	*a*_*it*_	**0.430**	**9.51**	**0.000**	**48.1**	**0.256**	**5.94**	**0.000**	**48.2**
	*a*_*it*_ *a*_*i*(*t*−1)_	**0.095**	**3.06**	**0.002**		**0.331**	**11.0**	**0.000**	
Exclude	*a*_*it*_	**0.431**	**9.53**	**0.000**	**49.4**	**0.259**	**6.01**	**0.000**	**49.4**
	*a*_*it*_ *a*_*i*(*t*−1)_	**0.091**	**2.89**	**0.004**		**0.323**	**10.6**	**0.000**	

The Stock-Yoga *F*-test 5 and 10 % critical values for two endogeneous predictors are approximately 21.0 and 11.0 respectively. Well-identified IV results appear in bold

The results under OLS and 2SLS are largely invariant to *y*_*i*(*t*−1)_. One explanation that might also account for the sensitivity of the results under the dynamic model to the status of *y*_*i*(*t*−1)_ is that *y*_*i*(*t*−1)_ functions like a surrogate for *a*_*it*_ *a*_*i*(*t*−1)_. Thus, if *a*_*it*_ *a*_*i*(*t*−1)_ is excluded from the model its effect in large part transmits through *y*_*i*(*t*−1)_. If *a*_*it*_ *a*_*i*(*t*−1)_ is included then the treatment effect heterogeneity is appropriately accounted for and *y*_*i*(*t*−1)_ has less impact.

Because *F*_StageI_ ≤2.3 (7.2) when *a*_*i*(*t*−1)_ is an endogeneous (exogeneous) predictor, implying weak identifiability, it is unwise to interpret the associated results. Attempts to strengthen identification by using *a*_*i*(*t*−2)_ **z**_*it*_ as an IV resulted in at most minor improvements (results not presented). Therefore, the key to identification of endogeneous (*a*_*it*_, *a*_*it*_ *a*_*i*(*t*−1)_) is the exclusion of *a*_*i*(*t*−1)_ from the outcome model. In other words, the required exclusion restriction is that there is no carryover effect of atypical use for individuals who switch to a conventional [β_3_ = 0 in (3)].

If *a*_*i*(*t*−1)_ is excluded from the outcome equation it makes little empirical difference whether or not it is used as an IV. The two endogeneous effects are well identified (F_StageI_ nearly 50) and their estimated effects are similar. However, as for the dynamic model, inclusion of *y*_*i*(*t*−1)_ led to the term involving *a*_*i*(*t*−1)_ (in this case *a*_*it*_ *a*_*i*(*t*−1)_) having a greater effect. With *y*_*i*(*t*−1)_ in the model the effect of *a*_*it*_ *a*_*i*(*t*−1)_ is 50 % greater than that of *a*_*it*_; absent *y*_*i*(*t*−1)_ the effect is one-quarter the size.

Because the estimated effects under 2SLS are significant and positive under the four well-identified scenarios, the evidence against the offsets hypothesis is again substantial. However, we cannot conclusively discern whether *a*_*i*(*t*−1)_ operates as a lagged effect or exclusively as a modifying effect distinguishing new and continuing atypical users.

## Discussion

In testing the offsets hypothesis we found that lagged treatment, *a*_*i*(*t*−1)_, has a profound impact on the results of the IVs analyses. Furthermore, the estimated coefficients were sensitive to the role of the lagged outcome, *y*_*i*(*t*−1)_.

In both the dynamic- and modified-treatment models, endogeneity of *a*_*i*(*t*−1)_ proved fatal for identification. In the dynamic treatment model (no modification by lagged treatment), the key to identifiability was inclusion of *a*_*i*(*t*−1)_ in the treatment selection equation for *a*_*it*_. In the modified-treatment model the key was exclusion of *a*_*i*(*t*−1)_ from the outcome model. In both cases, *a*_*i*(*t*−1)_ did not need to be used as an IV in order to obtain statistically significant results.

If *y*_*i*(*t*−1)_ was excluded then the effect of *a*_*it*_ tended to dominate that of any other treatment variable (*a*_*i*(*t*−1)_ in the dynamic model and *a*_*it*_ *a*_*i*(*t*−1)_ in the modified-treatment model) whereas if *y*_*i*(*t*−1)_ was included lagged treatment had substantially more influence. In all such models the estimated treatment effects were positive. The discrepancy of these results with the cross-sectional analysis may be due to the weakness of the IVs cross-sectionally, violations of the IV assumptions in the longitudinal models, model miss-specification, or combinations of these.

The only specification that supported the offsets hypothesis was the dynamic-treatment model when *a*_*i*(*t*−1)_ was an IV and *y*_*i*(*t*−1)_ a predictor. In this model, conditioning on *y*_*i*(*t*−1)_ appears justified since if *a*_*i*(*t*−1)_ has an effect on *y*_*i*(*t*−1)_ which in turn has an effect on *y*_*it*_, conditioning on *y*_*i*(*t*−1)_ is necessary for *a*_*i*(*t*−1)_ to be a valid IV (Fig. 2). Furthermore, it is possible that the inclusion of any term involving *a*_*i*(*t*−1)_ in the outcome equation leads to spurious effects. Therefore, it is plausible that the lone specification that obtained a negative estimate is the only valid specification! However, while use of *a*_*i*(*t*−1)_ as an additional IV is enticing, its validity relies on an exclusion restriction that is difficult to satisfy, especially when first differencing is used for estimation. Therefore, the results in which *a*_*i*(*t*−1)_ is not used as an IV appear more trustworthy.

An important new finding is that use of an atypical in the past year may have a carryover effect on mental health costs in the current year. Under the dynamic treatment model there was evidence that individuals who used an atypical in the prior year had greater mental health costs. The well-identified results for the modified-treatment model rely on the exclusion restriction that past treatment is irrelevant for individuals taking conventionals. Unfortunately the IVs are not powerful enough for all treatment variables to simultaneously be modeled as endogeneous. Therefore, it is not possible to make a reliable comparison between the dynamic- and modified-treatment models.

While longitudinal designs have clear advantages, the consequences of different assumptions must be carefully considered. Using DAGs to depict theoretical models may generate valuable insights into the variables thought to influence or confound the effects of interest, which in turn can lead to experimental designs and identification strategies that overcome concerns about unmeasured confounders. The sensitivity of the IV results for the offsets analysis to different assumptions about lagged treatment and lagged outcomes illustrates the importance of using external information to help specify the most appropriate model. In addition to using varied specifications to evaluate the sensitivity of results to different models and IV specifications, sensitivity analyses that evaluate the robustness to violations of the IV assumptions (Small 2007) may also be helpful.

Developed in the 1920’s (Wright 1928), IVs and their estimation methods are less well known among statisticians (Dowd 2011). However, the growing importance of and interest in health policy research and the need for IVs in this field is likely to foster increased methodological work and awareness of IVs in the future. In this paper the focus was longitudinal models, inspired in part by the fact that statistical methods developed for longitudinal data have widespread applicability [e.g., generalized estimation equations (Liang and Zeger 1986; Zeger and Liang 1986)]. IV methods for time-to-event and joint longitudinal-survival models are important areas for future research.

## Footnotes

1 Because risperidal was introduced prior to 1994 its approval status is constant in the sample and so cannot be used as an IV.

2 Because it is caused by both *A*_0_ and *U*, *Y*_0_ is known as a collider. In general, conditioning on colliders is problematic (VanderWeele 2011).

3 In DAG terminology, *U* is a common cause of *A*_0_ and *Y*_0_ and therefore a confounder of the effect of *A*_0_ on *Y*_0_.

4 Note that *A*_0_ is an IV conditional on *Y*_0_ and *X*. Therefore, the rationale for such a test is the same as that underlying the test of over-identifying restrictions (Small 2007). A significant finding would cast doubt on whether *Z* is a valid IV or suggest that some other assumption about the model is incorrect.

5 Similar arguments support use of other types of lagged predictors as IVs. For example, in spatial analyses of areal data and geographic variations, the average exposure at neighboring sites has been used as an IV (Land and Deane 1992; Anselin 1990).

6 As for area in the offsets analysis, interactions between the IVs and ***x***_*it*_ are candidate IVs.
